# Recalibration of the Multisensory Temporal Window of Integration Results from Changing Task Demands

**DOI:** 10.1371/journal.pone.0071608

**Published:** 2013-08-08

**Authors:** Pierre Mégevand, Sophie Molholm, Ashabari Nayak, John J. Foxe

**Affiliations:** The Sheryl and Daniel R. Tishman Cognitive Neurophysiology Laboratory, Children’s Evaluation and Rehabilitation Center, Departments of Pediatrics and Neuroscience, Albert Einstein College of Medicine, Bronx, New York, United States of America; University of Regensburg, Germany

## Abstract

The notion of the temporal window of integration, when applied in a multisensory context, refers to the breadth of the interval across which the brain perceives two stimuli from different sensory modalities as synchronous. It maintains a unitary perception of multisensory events despite physical and biophysical timing differences between the senses. The boundaries of the window can be influenced by attention and past sensory experience. Here we examined whether task demands could also influence the multisensory temporal window of integration. We varied the stimulus onset asynchrony between simple, short-lasting auditory and visual stimuli while participants performed two tasks in separate blocks: a temporal order judgment task that required the discrimination of subtle auditory-visual asynchronies, and a reaction time task to the first incoming stimulus irrespective of its sensory modality. We defined the temporal window of integration as the range of stimulus onset asynchronies where performance was below 75% in the temporal order judgment task, as well as the range of stimulus onset asynchronies where responses showed multisensory facilitation (race model violation) in the reaction time task. In 5 of 11 participants, we observed audio-visual stimulus onset asynchronies where reaction time was significantly accelerated (indicating successful integration in this task) while performance was accurate in the temporal order judgment task (indicating successful segregation in that task). This dissociation suggests that in some participants, the boundaries of the temporal window of integration can adaptively recalibrate in order to optimize performance according to specific task demands.

## Introduction

The age-old estimation of how far away a lightning strike is, involves counting the seconds between the flash of light and the subsequent clap or rumble of thunder. This method is based on the fact that, despite representing the same event, the different travelling speeds of light and sound cause the distant audiovisual stimuli to be perceived as two separate events. In daily life, however, multisensory stimuli are typically much closer to us. As a result, we remain unaware of the subtle differences in arrival time of sound and light, and thus perceive these stimuli as simultaneous. This ability to accommodate some degree of asynchrony between the senses to allow for the unitary perception of multisensory events has been termed the temporal window of integration (TWI) [1]–[3].

Just how long the TWI is, what are its tolerances and limits, and whether or not it is fixed or malleable, remain important open questions for multisensory researchers. The answers are not straightforward: the window depends not only on the asynchrony between the individual sensory signals, but also on many other factors, such as the specific sensory combinations under consideration [4], the intensity and duration of the inputs [5], their spatial separation [6], [7], and the complexity of the signals, such as audiovisual speech for example [8]–[11]. The TWI also varies significantly across individual participants [12], [13]. For simple audiovisual stimuli presented at the same location in space, estimates of the width of the TWI range from as little as 60 ms [7] to over 250 ms [14].

Along with these stimulus-related influences on the TWI, and perhaps more important from a clinical perspective, recent findings have suggested that the boundaries of the window are indeed plastic. For instance, providing participants with feedback on their performance in an audiovisual temporal order judgment (TOJ) task improves their ability to discriminate subtle asynchronies between the visual and the auditory stimuli, thus narrowing the window [15]. Furthermore, repeated exposure to a consistently offset audiovisual stimulus appears to shift the window towards the direction of the offset [16], [17]. Interestingly, this recalibration is larger if attention is directed toward the temporal features of the offset stimulus during the period of adaptation [18]. Attention influences the TWI on a shorter time scale, as demonstrated by the so-called prior entry effect: attending one sensory modality causes stimuli in that modality to be perceived as happening earlier; as a result, the window is shifted towards the attended modality [19], [20].

Here, we address a related question: do task demands have a short term influence on the TWI? The answer is relevant because task-related changes in perceptual decision making may represent a mechanism for performance optimization [21]. Additionally, studies have started to suggest that the TWI is abnormally long in neurodevelopmental disorders such as autism and dyslexia [22]–[24]. Understanding the underpinnings of the TWI would give us insight into the pathophysiology of these disorders, and may also point towards rehabilitative interventions.

We take advantage of the fact that two behavioral approaches to probing the TWI have been developed. The first one uses reaction time (RT) as an index of multisensory integration: if two unisensory stimuli fall inside the window, they are integrated, and this manifests as an acceleration of RT [25], [26]. In the second approach, participants are asked to report which stimulus they perceived first (TOJ task) or whether they perceived the stimuli as simultaneous or not, a similar but not identical task [27]. Here, it is assumed that when stimuli cannot be temporally discriminated, with behavioral performance below some specified criterion such as 75% accuracy [2], [28]–[30], they fall within the TWI.

We reasoned the following: in the TOJ task, participants had to try and discern subtle asynchronies between an auditory and a visual stimulus. Thus, if the TWI was at all influenced by task demands, it should be ‘set’ at its narrowest in order to optimize performance. By contrast, optimal performance in the RT task would entail widening the window to maximize multisensory facilitation. To test the influence of task goals on the TWI, we therefore assessed whether participants would be able to accurately discriminate the order of stimuli in the TOJ task while showing significant acceleration of responses in the RT task at the same stimulus onset asynchronies (SOA).

## Methods

### Ethics Statement

All procedures were approved by the ethical review board of Albert Einstein College of Medicine and were in accordance with the tenets set forth in the Declaration of Helsinki. All participants provided written informed consent before participating in this experiment.

### Participants

Fifteen participants (7 women; 13 right-handed) aged 18 to 40 years completed the experiment for a modest fee of $12/hour. They all reported normal or corrected to normal vision, normal audition, and the absence of any neurological or psychiatric condition.

### Stimuli and Procedure

Participants sat in a sound-attenuated darkened double-walled chamber (Industrial Acoustics Company Inc., Bronx, NY, USA). Stimulus presentation and response monitoring was performed using the Presentation 15.0 software (Neurobehavioral Systems, Albany, CA, USA). The visual stimulus was a red colored disc subtending 5.2 degrees of visual angle, situated 0.7 degrees above the fixation cross, presented through a CRT monitor for 10 ms (1 refresh cycle at 100-Hz refresh rate). The auditory stimulus was a 10-ms 1000-Hz sine wave with 3-ms linear rise and fall ramps, delivered through speakers positioned on top of the monitor, vertically aligned with the visual stimulus. Trials consisted of no stimulus presentation (catch trials), unisensory (visual alone or auditory alone) or multisensory stimulation, with the following SOAs: 0, +/−20, 40, 60, 80, 100, 120, 150, 200, 250, 300, 400 ms (negative SOAs indicated that the auditory stimulus preceded the visual stimulus). Catch trials were included to discourage anticipatory responses, to account for such responses in the RT data (see below) and because they will be used to compute event-related potentials in future studies [31]. The accuracy of timing was checked scrupulously with an oscilloscope and a photodiode. Participants responded using the left and right buttons of a computer mouse. Trials where the Presentation software reported timing uncertainties greater than 5 ms in stimulus presentation or response logging (average 0.98% of trials, range 0.58–2.12% across subjects and 0–8.64% across conditions) were excluded from further analysis.

In the RT task, participants had to respond as fast as possible to any stimulus, visual or auditory. When they were able to discern two discrete stimuli in a trial (e.g. an auditory stimulus 400 ms earlier than a visual stimulus), they were told to respond only to the first one. The inter-trial interval between bisensory pairings was randomly jittered between 1 and 3 s. Fast guesses were accounted for by using the distribution of responses to the catch trials, in a procedure known as “kill-the-twin” [32]–[34]. Briefly, for each response to the catch trials, a response of similar latency was removed from the distribution of responses to the visual stimulus and replaced by an infinitely long reaction time, as described by Gondan and Heckel [33]. In order to minimize the chances of erroneously rejecting the race model, a conservative approach to the “kill-the-twin” procedure was applied to the observed RT data, and a progressive approach was applied to the simulated RT data generated by the resampling procedure described below [33]. Misses were attributed infinitely long reaction times [35], [36]. The average occurrence of guesses to the catch trials was 3.94% across subjects (range 0–11.67%). The average occurrence of misses was 0.70% (range 0.2–3% across subjects and 0.15–5% across conditions).

In the TOJ task, participants had to signal which stimulus they perceived first, the auditory or the visual. The inter-trial interval between bisensory pairings was again randomly jittered between 1 and 3 s. Responses given earlier than 100 ms after the onset of the first stimulus in the trial were considered as false alarms and excluded from further analysis. If participants did not respond within 3 s of stimulus onset, an instruction screen reminded them which button corresponded to which response and waited for a response. Thus, there were no misses in this task.

The experiment consisted of two large blocks for each task. The order in which each subject underwent the blocks was randomly determined. Each block was subdivided into 26 mini-blocks of about 2 minutes, in which 30 trials were presented. Trial order was randomized, with the constraint that 30 repeats of each trial type were presented in a large block. Each trial type was thus presented in total 60 times for each task. Breaks were encouraged to maintain concentration and reduce fatigue.

### Data Analysis

Analyses were performed using MATLAB v7.11 (R2010b) with the Statistics toolbox (The Mathworks, Natick, MA, USA). Results are presented as mean and standard deviation. Comparisons between means were performed using Student’s t test and 2-factor ANOVA for independent samples.

### Reaction Time Task: Violation of the Race Model

The analysis for the RT task identified multisensory integration by assessing whether RT distributions violated the race model [37]. This model places an upper limit on the acceleration of reaction time to a multisensory stimulus that can be expected due to probability summation of responses to unisensory stimuli. For any post-stimulus latency t, the race model holds if the response probability to a multisensory stimulus is no larger than the summed response probabilities to unisensory stimuli: P_AV_(t) ≤P_A_(t)+P_V_(t). For SOAs other than 0, t is replaced by t+SOA [26], [38].

To test for race model violation, we first computed the cumulative distribution functions (CDFs) of reaction times to multisensory and summed unisensory stimuli, using published algorithms [39], [40]. We then collapsed the differences between the time points at which the CDFs reached percentiles 10, 15, 20 and 25 for the multisensory versus the summed unisensory distributions into a single statistic: D = ∑[CDF_AV_(p) – CDF_A+V_(p)]. This collapsing avoided the accumulation of type I statistical error due to testing at multiple percentiles while retaining adequate power [26], [40]–[42]. Values of D lower than zero indicate violation of the race model.

To test the statistical significance of observed values of D at the single-participant level, we used a resampling procedure to simulate the distribution of D* under the null hypothesis that the race model holds [26], [41]. In each iteration of the procedure, we built a simulated CDF for the summed unisensory stimuli by randomly sampling with replacement a reaction time for one sensory modality and pairing it with a reaction time for the other modality. In order to maximize the negative correlation and thus the redundancy gain between auditory and visual reaction times, the pairing was not random; rather, the response at percentile P for a given modality was paired with the response at percentile 1– P for the other modality, and the fastest reaction time was added to the simulated CDF. This procedure makes it harder to violate the race model and contributes to the conservativeness of the test [26], [41]. We then computed D* as above, replacing the observed CDF by the simulated one. The procedure was repeated 1000 times. The observed value of D was then compared to the distribution of D* using a one-sided test with the significance level set at 0.05.

Statistical testing was performed separately for each SOA. In the case that a participant displayed a discontinuous pattern of race model violation (e.g. violation with SOAs of −40 to +80 ms, no violation at +100 ms, and violation again at +120 ms), we restricted the definition of the TWI to the contiguous SOAs with significant race model violations that were around physical simultaneity or closest to it (e.g. −40 to +80 ms). This compensated partly for the repetition of statistical testing.

In order to assess the performance of our participants on the RT task at the group level, we used a sign permutation test to evaluate the statistical significance of observed values of D across individuals [41]. Under the null hypothesis that the race model holds, there is no systematic difference between CDF_AV_ and CDF_A+V_, and values of D at each SOA across participants can be indifferently positive or negative. At each SOA, we therefore randomly reassigned each participant’s value of D to be either positive or negative and summed the D* values across participants. All possible sign permutations were used to build the distribution of ∑D* under the null hypothesis. We then compared the observed ∑D to the distribution of ∑D* using a one-sided test with the significance level alpha set at 0.05. This group-level analysis was used only for illustrative purposes; the main results and conclusions of the present article are based on individual-participant-level analyses.

### Temporal Order Judgment Task: Bayesian Analysis for Logistic Regression

**Figure 1:**
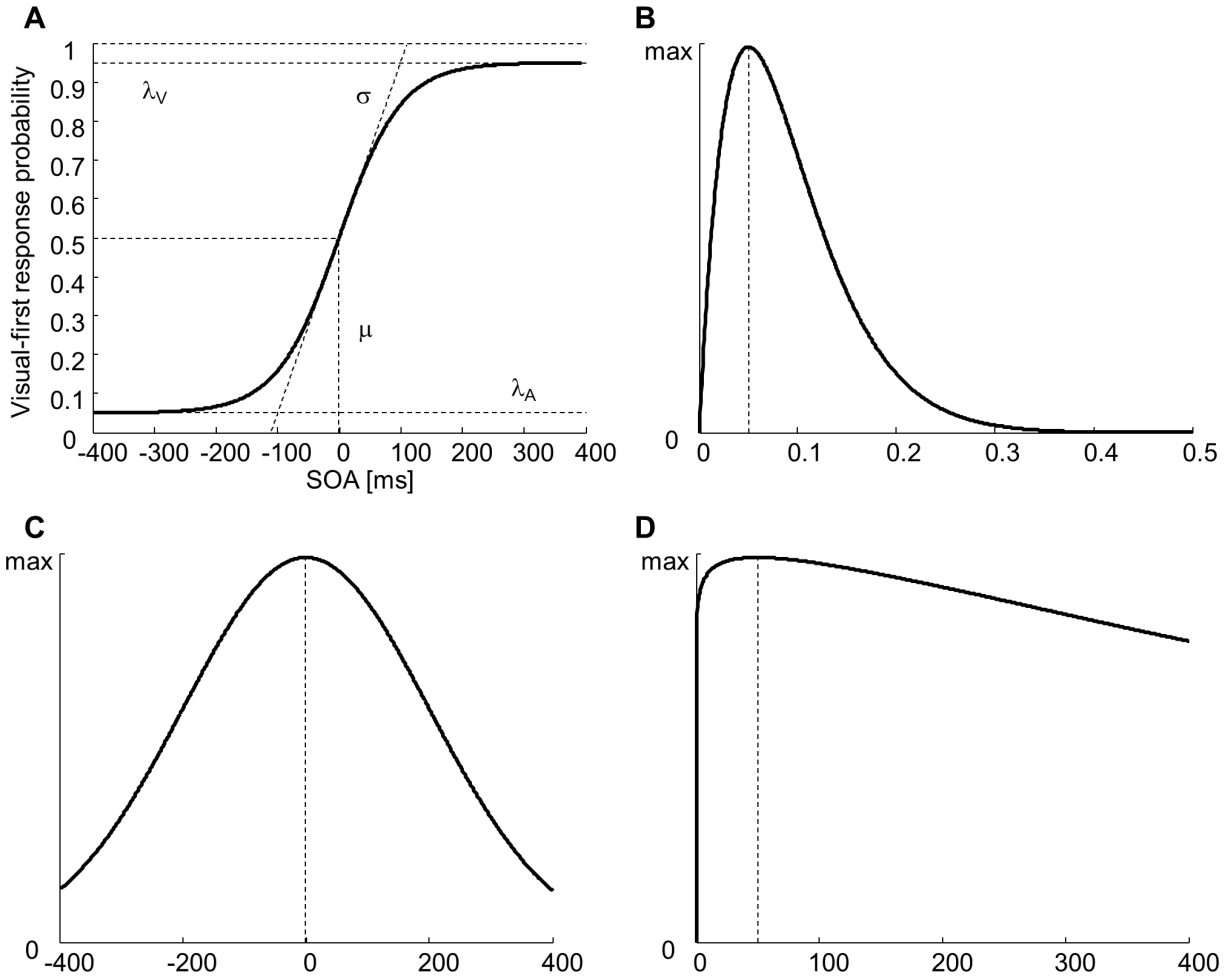
Parameters of the psychometric function and priors for the Bayesian analysis. **A.** The parameters that define the logistic function are illustrated: λ_A_, lapse rate when the auditory stimulus precedes the visual stimulus by a long interval (large negative values of the stimulus onset asynchrony, SOA); λ_V_, lapse rate when the visual stimulus precedes the auditory one (large positive values of the SOA); μ, location parameter; σ, shape parameter. In order to illustrate how the prior distributions selected for each parameter (illustrated in the following panels) affect the shape of the logistic function, the numerical values of the parameters were set to the maximum of their prior distribution. **B.** The beta distribution used as a prior for the lapse rates λ_A_ and λ_V_. The maximum prior probability corresponds to λ = 0.05. **C.** The normal distribution used as a prior for the location parameter μ. The maximum prior probability corresponds to μ = 0. **D.** The gamma distribution used as a prior for the shape parameter σ. The maximum prior probability corresponds to σ = 50.

For the TOJ task, the analysis identified thresholds for above-chance performance. We expressed participants’ responses as the proportion of “visual first” responses for each SOA (unisensory trials were not included in the analysis). We then used Bayesian analysis to perform logistic regression in order to fit a psychometric function to the data, as previously described [43], [44]. An advantage of Bayesian analysis over maximum likelihood estimation procedures is that the former yields more accurate point estimates and more accurate and tighter confidence intervals for the parameters of the psychometric function [43]. Our psychometric function took into account lapses [45]: Ψ(x; μ, σ, λ_A_, λ_V_) = λ_A_+(1– λ_A_ – λ_V_) F(x; μ, σ), where λ_A_ and λ_V_ are the lapse rates for large SOAs with the auditory and visual stimulus leading, respectively, and F(x; μ, σ) is the logistic cumulative distribution function with μ and σ as the location and shape parameters (Figure 1A). The threshold for above-chance performance on the “auditory-first” side of the psychometric function was defined as the time point where performance was halfway between the lapsing rate λ_A_ and 0.5, that on the “visual-first” halfway between 0.5 and 1- λ_V_. These thresholds correspond to 75% correct responses if there are no lapses [45].

In Bayesian inference, the posterior distribution of the parameters is defined as a function of their prior distributions P(Θ) and of the likelihood function:
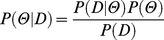
Where Θ is the set of parameters and D is the observed data. Here, the likelihood function is defined using the probability mass function of the binomial distribution [44]:



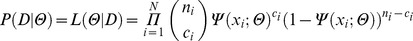
Where i = 1,…,N represents the SOAs, x_i_ is each SOA, n_i_ is the number of trials at each SOA, c_i_ is the number of visual-first responses at each SOA, and Ψ(x_i_; Θ) is the logistic function. P(D) is a normalizing constant defined as:



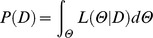



The prior probability distributions of the parameters P(Θ) were selected by taking into account generally accepted assumptions [43], [44]. The lapse rates necessarily lie between 0 and 1, are unlikely to be exactly 0 (i.e. no lapses), and are expected to be relatively low. We integrated these assumptions by selecting a beta prior with shape parameters α = 2 and β = 20 (Figure 1B). The location parameter of the logistic function is expected to be roughly centered on physical simultaneity, but varies significantly across participants [11], [13]. We therefore chose a normally distributed prior with a mean of 0 and standard deviation of 200 (Figure 1C). The shape parameter of the logistic function must be larger than 0; otherwise, we had few assumptions about this parameter and therefore chose a gamma prior with shape parameters κ = 1.05 and θ = 1000, yielding a relatively uniform probability density function (Figure 1D).

Because the integral in the normalizing constant P(D) is analytically intractable, the posterior distribution of the parameter set can only be approximated. We here used slice sampling [46], a type of Markov chain Monte Carlo algorithm, to generate pseudo-random samples from the posterior distribution. Slice sampling is based on the premise that one can sample from a distribution by sampling uniformly from the region under the plot of its density function. Slice sampling is able to sample from an arbitrary density function known only up to a constant, as is the case here. An advantage of slice sampling is that, contrary to other Markov chain Monte Carlo algorithms such as the Metropolis-Hastings rule used in [43], only the scaled posterior distribution must be specified. In the univariate case, the algorithm goes through the following steps: (1) Assume an initial value of x, x_0_, within the domain of f(x). (2) Draw an auxiliary value y uniformly from the interval (0, f(x_0_)); y defines a “slice” of the distribution defined as S = [x: y<f(x)]. (3) Find an interval I = (L, R) that contains all or much of the “slice” S. (4) Draw a new point x_1_ within this interval. (5) Repeat steps 2 to 4 with x_1_ until the number of desired samples is reached. The slice sampler algorithm is implemented in the MATLAB Statistics Toolbox (function slicesample). Because the first iterations of the slice sampler tend to yield non-stationary outputs, we rejected the first 100 iterations. Additionally, in order to minimize the autocorrelation between samples from adjacent iterations, we thinned out the output of the slice sampler by accepting only every tenth value. We then generated 2000 samples of the posterior distribution of the parameters.

For single-participant analysis, we used these 2000 samples to compute 2000 psychometric functions and obtain 2000 estimates of both the “auditory-first” and “visual-first” thresholds. Each individual’s “auditory-first” threshold was then estimated as the 5^th^ percentile of the obtained threshold samples, so that the actual threshold had a probability of 0.05 of being more negative (i.e. further away from simultaneity on the “auditory-first” side) than the estimated value. Similarly, the “visual-first” threshold was estimated as the 95^th^ percentile of the samples for that threshold, so that the probability that the actual threshold was more positive (i.e. further away from simultaneity on the “visual-first” side) was 0.05. These estimates can be considered conservative, representing the widest boundaries of the TOJ-defined TWI in each participant.

Four participants did not reach the threshold level of performance on one or the other side of the psychometric function for the TOJ task and were excluded from further analysis (see [7], [47], [48] for a similar approach to excluding participants who were unable to perform the TOJ task).

In order to assess the performance of our participants on the TOJ task at the group level, we used each individual’s medians of the 2000 samples from the posterior distribution as estimates of the individual parameters [43], averaged the individual parameter estimates to compute the group psychometric function, and extracted the thresholds from that function. This group-level analysis was used only for illustrative purposes; the main results and conclusions of the present article are based on individual-participant-level analyses.

### Comparing the Temporal Window of Integration Across Tasks

Our hypothesis states that the TWI is recalibrated by task demands if we observe successful segregation of sensory inputs (i.e. above-chance performance) in the TOJ task at the same SOA where there is successful integration of inputs (i.e. race model violation) in the RT task. Because race model violation is a conservative assessment of multisensory integration [26], [41], our estimate of the RT-defined TWI errs on the side of being too narrow. Conversely, because we used the widest possible confidence estimates for the thresholds of the TOJ psychometric curve (cf. above), our estimate of the TOJ-defined TWI tends to be too wide. With these considerations in mind, markedly above-chance performance in the TOJ task at a SOA where there is race model violation in the RT task can be considered a robust index of task demand-induced TWI recalibration. Note that the reverse situation (i.e. chance-level performance in the TOJ task and no race model violation in the RT task) is not taken to indicate TWI recalibration, since the absence of evidence for race model violation (and hence multisensory integration) does not imply evidence of its absence.

### Data Sharing Statement

The complete datasets (reaction time and temporal order judgment data) from all 11 subjects retained for final analysis are provided as Supplemental Information (Dataset S1).

## Results

**Figure 2:**
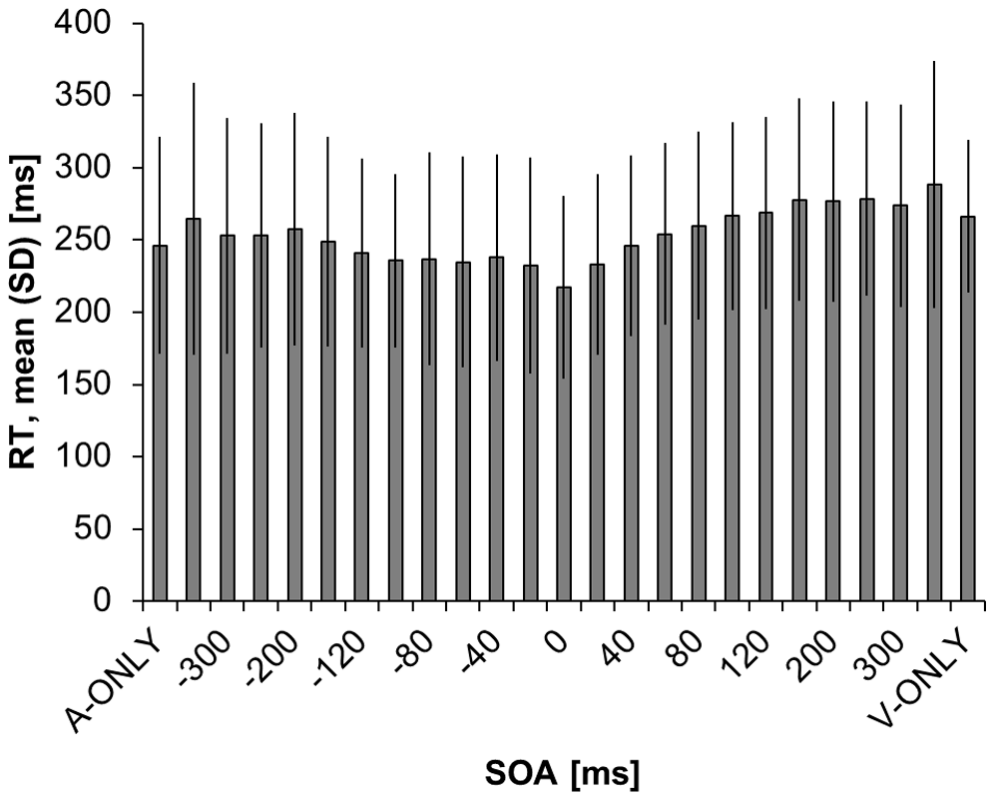
Participants’ performance on the reaction time task at the group level. The group performance on the reaction time (RT) task is plotted as the mean and standard deviation of reaction times as a function of the stimulus onset asynchrony (SOA). A-only and V-only indicate reaction times to unisensory, auditory- and visual-only trials respectively. The complete list of SOAs is provided in the Methods section.

**Figure 3:**
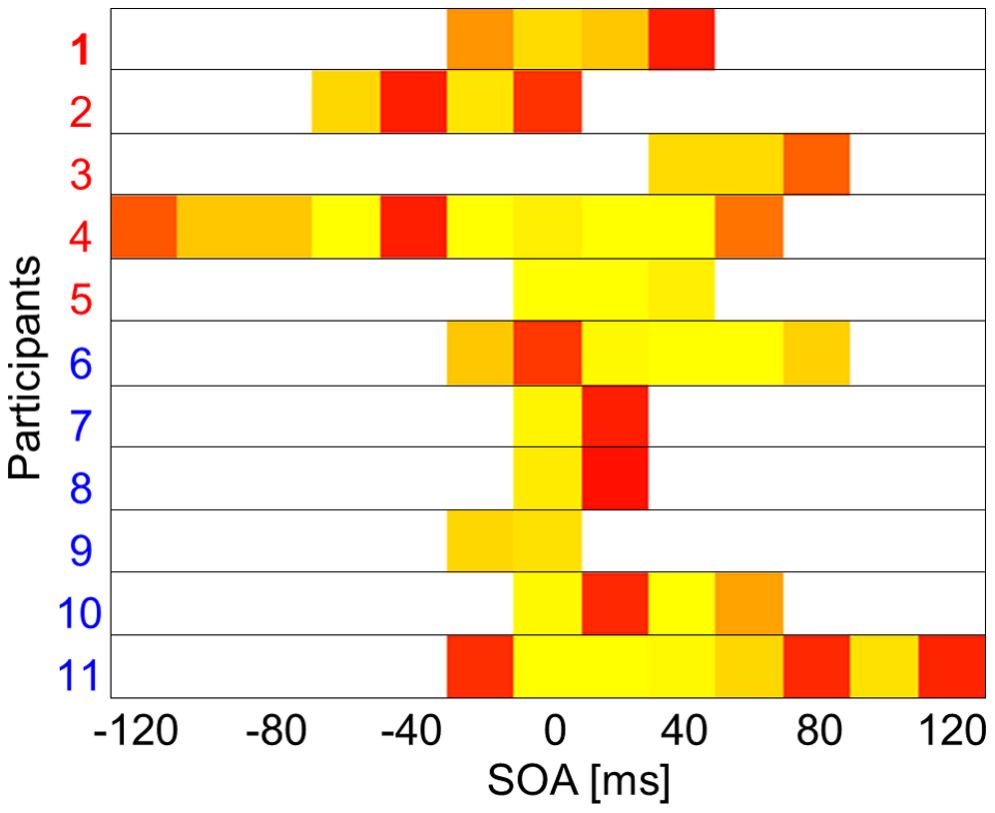
Race model violation in the reaction time task at the individual participant level. In this plot, each participant is represented by one line and each stimulus onset asynchrony (SOA) by one column. The p value for violation of the race model is color-coded for each participant and each SOA. Red color indicates p values closer to 0.05, yellow color indicates p values closer to 0. White color indicates that the race model could not be confidently rejected. Participant 1 (bold number) is the exemplary participant illustrated in Figure 5. None of the subjects displayed race model violation at SOAs beyond −120 ms and beyond +120 ms. Participants 1 to 5 (red numbers) displayed task-induced TWI malleability, whereas Participants 6 to 11 (blue numbers) did not.

**Figure 4:**
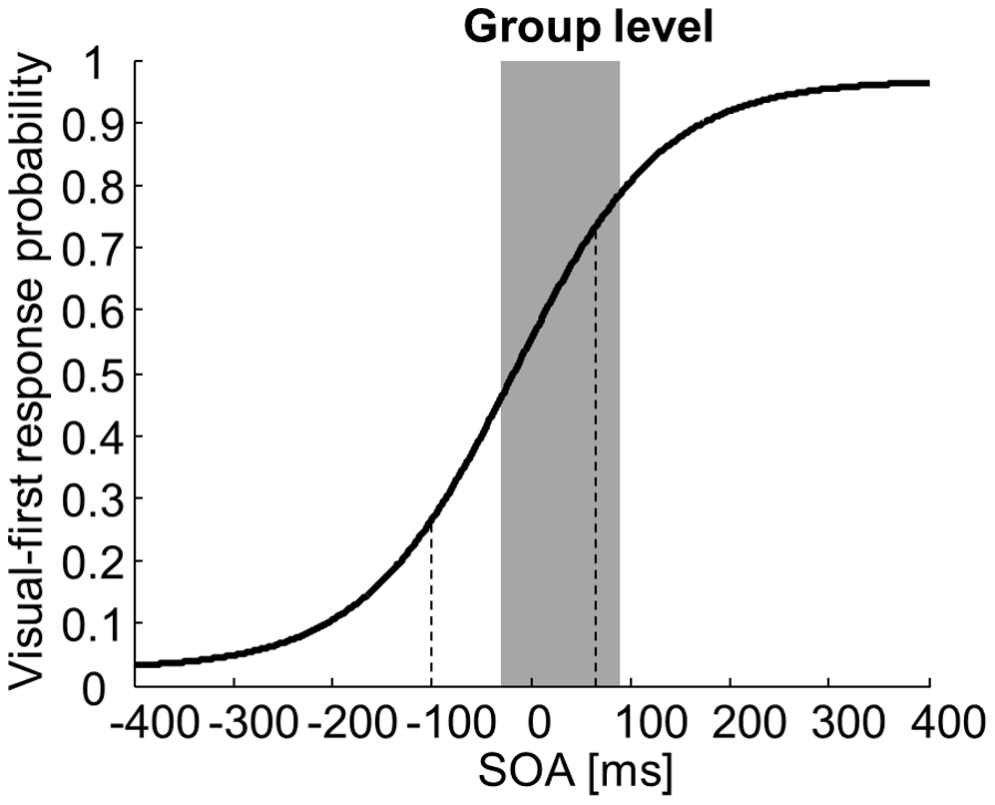
Participants’ performance at the group level. The group performance on the temporal order judgment (TOJ) task is plotted as the proportion of “visual-first” responses as a function of the stimulus onset asynchrony (SOA, bold curve). The dashed lines represent the thresholds for above-chance performance on the TOJ task. The grayed area represents the SOAs where significant violation of the race model was observed on the reaction time task.

The participants’ performance on the RT task is presented in Figure 2. Multisensory integration in the RT task, assessed using the race model at the individual participant level, is illustrated in Figure 3. When the visual and auditory stimuli were presented simultaneously (SOA of 0 ms), all but one participant displayed race model violation, indicating multisensory integration. Race model violation was more common when the visual stimulus led the auditory stimulus (positive SOAs), similar to previous reports [26]. At the group level, the TWI defined by the RT task ranged from SOAs of −20 to +80 ms (Figure 4).

Results of the participants’ performance on the TOJ task at the group level are presented in Figure 4. The group-level TWI defined by the TOJ task ranged from −101 to +65 ms. The fact that the “visual-first” TWI boundary defined by the TOJ task is slightly closer to physical simultaneity than that defined by the RT task suggests that, on average, participants may adapt the width of their TWI to optimize performance.

**Figure 5:**
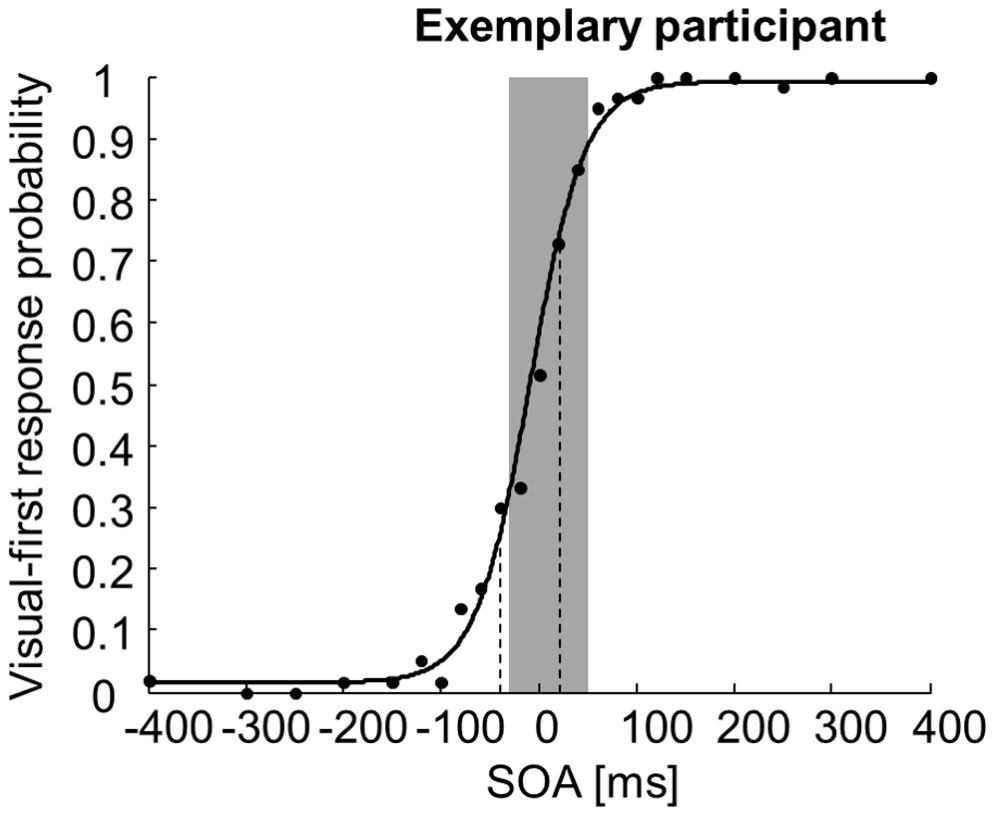
Results for an exemplary individual participant. The participant’s performance on the temporal order judgment (TOJ) task is plotted as the proportion of “visual-first” responses as a function of the stimulus onset asynchrony (SOA, filled circles). The psychometric function fitted onto these data is plotted as a continuous line. Dashed lines indicate the thresholds for above-chance performance. The grayed area represents the SOAs where significant violation of the race model was observed on the reaction time task.

Analyzing the performance of individual participants confirmed that some displayed TWI malleability in response to task demands. In 5 out of 11 participants, there were SOAs where performance was above chance on the TOJ task despite significant race model violation on the RT task. Specifically, in 4 participants, the “visual-side” TWI boundary defined by the TOJ task was closer to physical simultaneity than that defined by the RT task. One exemplary participant performed markedly above chance on the TOJ task at an SOA of +40 ms while displaying significant violation of the race model on the RT task at this SOA (Figure 5). In one additional participant, the “auditory-side” TWI boundary defined by the TOJ task was closer to physical simultaneity than that defined by the RT task. The average performance of these 5 participants on the TOJ task at these SOAs where they displayed race model violation averaged 86% (range 75–98.33%), indicating clearly above-chance performance.

**Figure 6:**
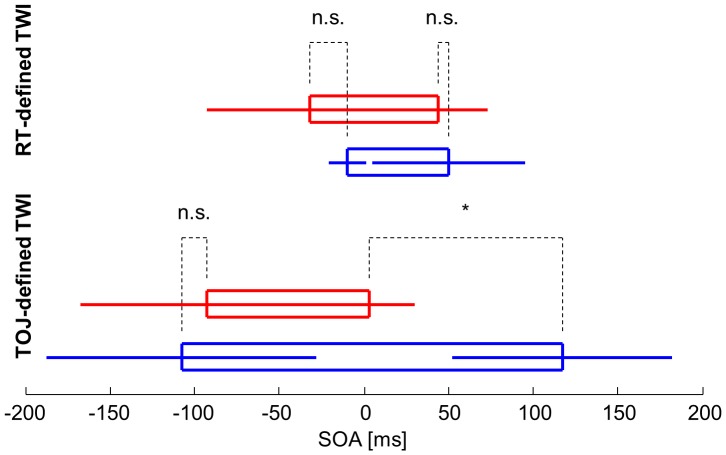
The reaction time- and temporal order judgment-defined temporal window of integration. Means and standard deviations of the boundaries of the temporal window of integration (TWI) defined by the reaction time (RT) and temporal order judgment (TOJ) tasks for the participants who displayed task demand-induced TWI malleability (red, n = 5) and for those who did not (blue, n = 6). n.s. not significant, *p<0.05.

In the 5 participants where performance suggested task demand-induced TWI malleability, the width of the TOJ-defined window was significantly narrower than in the other 6 participants (96 (57) ms vs. 225 (76) ms; t = −3.119, p = 0.0123), whereas the width of these 5 participants’ RT-defined TWI did not differ from that of the others (76 (59) ms vs. 60 (51) ms; t = 0.4849, p = 0.6393) (Figure 6). This suggests that these 5 participants did not merely have a narrower TWI regardless of the task they performed. In addition, the “visual-first” boundary of the TOJ-defined TWI in the 5 participants was closer to physical simultaneity than in the others (+3 (12) ms vs. +117 (27) ms; t = −3.6424, p = 0.0054), whereas the “auditory-first” boundary was not (−93 (33) ms vs. −108 (32) ms; t = 0.3248, p = 0.7528), arguing against a global shift of the window towards the “auditory-first” side in these participants. Neither boundary of the RT-defined TWI was different between the subsets of participants.

We also performed a 2-factor ANOVA on the TWI widths with task (RT vs. TOJ) as one factor and group (TWI-induced malleability vs. no malleability) as the other. There was a main effect of the task factor: the mean RT-defined TWI was narrower than the mean TOJ-defined TWI (67 (52) vs. 166 (94) ms; F = 14.1, p = 0.0014), as expected from previous findings that race model violation on the RT task occurs over a narrower range of SOAs than chance-level performance on the TOJ task [7], [26]. There was also a main effect of the group factor: the TWI was overall narrower across tasks in the participants who displayed task-induced TWI malleability than in those who did not (86 (56) vs. 143 (106) ms; F = 4.57, p = 0.0465). Most importantly, there was a significant interaction between the 2 factors (F = 7.52, p = 0.0134), confirming our finding that task-induced TWI malleability was due to a narrower TOJ-defined TWI in the participants displaying malleability, without any difference in the RT-defined TWI.

## Discussion

In this study, we measured the temporal window of multisensory integration for both reaction time and temporal order judgment tasks, using audiovisual stimuli with varying stimulus onset asynchronies, in the same participants. Our main finding is that there are participants who display significant race model violation on the RT task at SOAs at which they perform well above chance on the TOJ task. This suggests that these individuals are able to adaptively modify the boundaries of their TWI in order to optimize performance depending on task demands. More than half of the participants however did not demonstrate task-related modulation of the TWI, indicating that this ability is not ubiquitous with the experimental design used here.

There have previously been few studies where RT and TOJ performance to audiovisual stimuli were compared in the same participants [29], [30]. In these studies, the difference between RT to unisensory stimuli was compared to the point of subjective simultaneity of the TOJ task as estimates of intersensory differences in perceptual latency. Discrepancies in the results given by the two approaches have been thoroughly discussed [21], [49]–[51]. However, these studies focused on a point estimate of the position of the TWI rather than on its width and boundaries, as is the case here, and did not assess RT to multisensory stimuli. We are therefore left to compare our results with studies where the RT- and TOJ-defined TWIs were assessed separately.

Regarding the RT task, in contrast to the widespread use of race model violation to identify audiovisual integration using synchronous stimuli (see e.g. [37], [52]–[54]), the effect of systematically varying the SOA has been much less studied. Miller observed race model violation between 0 and +167 ms in one participant and at +67 and +100 ms in another one (SOAs ranged from −167 to +167 ms in that study) [26], while Diederich and Colonius [25] reported race model violation between 0 and +50 ms in a group of 4 participants (no other SOA was tested in that study). These results are in agreement with ours, and also illustrate the large inter-individual variability of the RT-defined TWI. Our group TOJ psychometric function is also similar to previous studies that used comparable experimental settings [5], [7], [11]. Again, large inter-individual variability was reported in simultaneity tasks [12], [13]. This variability warrants the analysis of the performance of individual participants in addition to much more common traditional group-level analyses.

We observed TWI malleability in response to task demands only in a subset of our participants. We speculate that the potential for such malleability is in fact present in everyone, but that our experimental design did not reveal it in some individuals. These individuals had on average a wider TOJ-defined TWI (see Figure 4). It has been shown that the TWI can be narrowed by training participants in a simultaneity task and giving them feedback on their performance, and that the effect of training is larger in individuals with an initially larger window [15]. Thus, it should be possible to narrow the TOJ-defined TWI in those of our participants who have a larger window at baseline by training them on the TOJ task. On the other hand, Miller reported no effect of experience on the RT-defined TWI despite the expected accelerations of RT due to practice [26], [37]. Therefore, training on the RT task is not expected to either narrow or widen the RT-defined window. Altogether, we speculate that adequate training on our tasks should lead most, if not all, participants to display TWI malleability. This should clearly be tested in future studies.

Why our participants have a narrower or wider TOJ-defined window at baseline may be explained in part by varying levels of expertise in discriminating subtle spatiotemporal features of naturalistic multisensory stimuli in every-day life. An example is provided by people who play action video games, where such fine discrimination is necessary for optimal performance. Video game players have a narrower TWI on audiovisual TOJ and simultaneity tasks, the magnitude of the effect correlating with the amount of video game expertise [14]. In addition, several hours of video game practice in non-players reduces both backward visual masking and the attentional blink [55], [56], indicating that training on video games does improve temporal discriminative performance, at least in the visual modality. Improved temporal discriminative abilities are also found in musical experts: conductors perform more accurately than musically untrained controls on an auditory TOJ task [57], and drummers are better able to detect asynchrony in an audiovisual point-light drumming movie [58]. Varying degrees of expertise in these and other situations may thus account for part of the inter-individual variability in the baseline width of the TOJ-defined TWI.

In our experiment, TWI malleability in response to task demands occurred more often on the “visual-first” side of the window than on its “auditory-first” side. Others also observed asymmetric effects of experimental manipulations on the TWI: training on a simultaneity task narrowed the “visual-first” but not the “auditory-first” side of the window [15], [59], and exposure to temporally offset audiovisual stimuli was more effective at recalibrating the window towards the offset when the visual stimulus led than when the auditory one did [16]. We speculate that this larger capacity of the “visual-first” side for malleability represents an adaptation to the fact that, in natural settings, the sound emitted by a multisensory event can never physically precede the visual signal. Interestingly, the TWI is wider in ten- and eleven-year-old children than in adults; it extends more in the “auditory-first” direction and is thus symmetrical [60]. The “auditory-first” side of the TWI thus appears to narrow over a protracted developmental period, putatively because of repeated ecological exposure to “visual-leading” stimuli and absence of exposure to “auditory-leading” stimuli. Similarly, we suggest that the capacity for TWI plasticity is initially symmetric in children, and then dwindles on the “auditory-first” side later during development for want of exposure to “auditory-leading” stimuli. This idea could be explored, for instance, by testing whether the abovementioned experimental manipulations would have larger effects on the “auditory-first” side of the TWI in children than in adults.

Whether the RT and TOJ tasks used here engage the same internal detection and decision mechanisms remains uncertain. In the visual modality, recent psychophysical evidence suggests that the same processes are indeed set into play by both tasks, at least at the initial stages of processing [61], [62]. What, then, could the neural substrates for the multisensory TWI be? Pioneering work identified a TWI in the multisensory responses of single neurons in the superior colliculus of cats [63] and monkeys [64]. In humans, the detection of audiovisual synchrony activates a large-scale network including the posterior parietal, superior temporal, prefrontal and insular cortices in addition to early visual and auditory areas and the posterior thalamus and superior colliculus [65]–[72]. TOJ training-induced narrowing of the TWI was associated with a reduction in fMRI responses in the posterior superior temporal cortex and early visual and auditory areas, implicating these areas as key nodes for plasticity [59]. Although it is currently unknown how differing task demands influence the neural underpinnings of the TWI, it is reasonable to assume that one or several of the abovementioned areas will be affected.

EEG studies have revealed that the integration of synchronous audiovisual stimuli begins at very early post-stimulus latencies and thereafter proceeds over the next several hundred milliseconds [52], [69], [73]–[75]. Interestingly, it was shown in a simultaneous auditory-somatosensory RT task that the earliest multisensory integrative effect (taking place between 40 and 84 ms post-stimulus) was only present in those trials where reaction times were faster and the race model was significantly violated [76], [77]. Electrical source imaging localized this early integrative effect in general vicinity of the posterior superior temporal cortex. Coming back to our experiment, we hypothesize that a similar early modulation of activity in the posterior superior temporal cortex would index successful multisensory integration at SOAs where race model is violated. It would then be extremely informative to assess whether that early effect is also present at the same SOA in the TOJ task in participants who perform well above chance. If it were to be observed, then it may represent a relatively automatic, bottom-up index of stimulus coincidence with no direct bearing on the ultimate behavioral performance. If, on the other hand, it were selectively abolished in the TOJ task, it would imply that the earliest stage of multisensory integration is already subject to top-down, cognitive influences. Neurophysiological studies using the experimental design proposed here will be needed to answer this question.

### Conclusions

In this study, we have demonstrated that the temporal window of multisensory integration is malleable: its boundaries can change depending on the particular task being performed. The experimental design presented here may prove useful to later examine how cognitive factors influence the neural dynamics of multisensory integration.

## Supporting Information

Dataset S1 **Complete reaction time and temporal order judgment data.** The dataset consists of one MATLAB (.mat) data file including one data structure, itself made up of 11 substructures (one per participant, with the numbers corresponding to those used elsewhere in the article). Each participant’s reaction time data are contained in a 26-by-60 RT cell array. The first dimension represents reaction times for each of the SOA, always in the same order: (1) Catch trials (no stimuli), (2) Auditory-only trials, (3) SOA −400 ms, (4) SOA −300 ms, (5) SOA −250 ms, (6) SOA −200 ms, (7) SOA −150 ms, (8) SOA −120 ms, (9) SOA −100 ms, (10) SOA −80 ms, (11) SOA −60 ms, (12) SOA −40 ms, (13) SOA −20 ms, (14) SOA 0 ms, (15) SOA +20 ms, (16) SOA +40 ms, (17) SOA +60 ms, (18) SOA +80 ms, (19) SOA +100 ms, (20) SOA +120 ms, (21) SOA +150 ms, (22) SOA +200 ms, (23) SOA +250 ms, (24) SOA +300 ms, (25) SOA +400 ms, (26) Visual-only trials. The second dimension represents reaction times for each trial of a given SOA. The total number of reaction times per SOA may be below 60, reflecting the exclusion of trials with Presentation software timing uncertainties above 5 ms. Reaction times are given in milliseconds. Trials where no response was given are coded as Inf (infinitely long reaction time). The reaction time data have undergone the kill-the-twin procedure described in the Methods. Each participant’s temporal order judgment data are contained in a 23-by-3 TOJ numeric array. The first column contains the SOA used in that task. The second column contains the number of trials where the participant gave a “visual-first” response for the corresponding SOA. The third column contains the total number of trials for the corresponding SOA. That number may be below 60, reflecting the exclusion of trials with Presentation software timing uncertainties above 5 ms, as well as those where response latency was below 100 ms.
(MAT)
